# Modulation of u-PA, MMPs and their inhibitors by a novel nutrient mixture in adult human sarcoma cell lines

**DOI:** 10.3892/ijo.2013.1934

**Published:** 2013-05-09

**Authors:** M. WAHEED ROOMI, TATIANA KALINOVSKY, ALEKSANDRA NIEDZWIECKI, MATTHIAS RATH

**Affiliations:** Dr Rath Research Institute, Santa Clara, CA 95050, USA

**Keywords:** fibrosarcoma, chondrosarcoma, liposarcoma, synovial sarcoma, uterine leimyosarcoma, u-PA, MMP-2 and MMP-9, TIMP-2, PMA, nutrient mixture

## Abstract

Adult sarcomas are highly aggressive tumors that are characterized by high levels of matrix metalloproteinase (MMP)-2 and -9 secretions that degrade the ECM and basement membrane, allowing cancer cells to spread to distal organs. Proteases play a key role in tumor cell invasion and metastasis by digesting the basement membrane and ECM components. Strong clinical and experimental evidence demonstrates association of elevated levels of u-PA and MMPs with cancer progression, metastasis and shortened patient survival. MMP activities are regulated by specific tissue inhibitors of metalloproteinases (TIMPs). Our main objective was to study the effect of a nutrient mixture (NM) on the activity of u-PA, MMPs and TIMPs in various human adult sarcomas. Human fibrosarcoma (HT-1080), chondrosarcoma (SW-1353), liposarcoma (SW-872), synovial sarcoma (SW-982) and uterine leimyosarcoma (SK-UT-1) cell lines (ATCC) were cultured in their respective media and treated at confluence with NM at 0, 50, 100, 250, 500 and 1,000 *μ*g/ml. Analysis of u-PA activity was carried out by fibrin zymography, MMPs by gelatinase zymography and TIMPs by reverse zymography. Fibrosarcoma, chondrosarcoma, liposarcoma and leiomyosarcoma cancer cell lines expressed u-PA, which was inhibited by NM in a dose-dependent manner. However, no bands corresponding to u-PA were detected for synovial sarcoma cells. On gelatinase zymography, fibrosarcoma, chondrosarcoma, liposarcoma and synovial sarcoma showed bands corresponding to MMP-2 and MMP-9 with enhancement of MMP-9 with PMA (100 ng/ml) treatment. Uterine leiomyosarcoma showed strong bands corresponding to inactive and active MMP-9 and a faint band corresponding to MMP-9 dimer induced with PMA treatment, but no MMP-2 band. NM inhibited their expression in a dose-dependent manner. Activity of TIMPs was upregulated by NM in all cancer cell lines in a dose-dependent manner. Analysis revealed a positive correlation between u-PA and MMPs and a negative correlation between u-PA/MMPs and TIMPs. These findings suggest the therapeutic potential of NM in treatment of adult sarcomas.

## Introduction

According to the American Cancer Society, about 11,280 new soft tissue sarcomas would be diagnosed in 2012 (6,110 cases in males and 5,170 cases in females) and 3,900 Americans (2,050 males and 1,850 females) were expected to die of soft tissue sarcomas (1). The most common types of sarcoma in adults are malignant fibrous histiocytoma/fibrosarcoma, liposarcoma (a malignancy of fat cells), and uterine leiomyosarcoma (1). Chondrosarcoma, a malignancy of cartilaginous origin, primarily affects the cartilage cells of femur, arm, pelvis, knee and spine. Forty percent of primary bone cancers are chondrosarcomas (2). Fibrosarcoma, a rare form of cancer (4% of bone cancers), is an aggressive and highly metastatic cancer of the connective tissue that primarily develops in metaphases of long tubular bones and affects both children and adults (2,3). Primary cancers of bones account for less than 0.2% of all cancers. Synovial sarcoma, also a rare soft tissue cancer (accounting for less than 5–10% of all soft tissue sarcomas) develops in the synovial membrane of the joints, most frequently occurs in the lower limbs, but can also occur in the trunk and head/neck (4). Poor prognosis is attributed to both the aggressive metastatic spread characteristic of these cancers and the lack of efficacy in current treatment modalities to prevent or counteract tumor progression. The overall relative 5-year survival rate of people with soft tissue sarcomas is around 50% according to statistics from the National Cancer Institute (1). The corresponding 5-year relative survival rates were: 83% for localized sarcomas (56% of soft tissue sarcomas were localized when they were diagnosed); 54% for regional stage sarcomas (19% were in this stage); 16% for sarcomas with distant spread (16% were in this stage) (1).

Metastasis occurs secondary to cancer cell detachment from the primary tumor, invasion through degraded basement membrane into the surrounding stroma, and entry into and transport through the vascular or lymphatic system to distal sites such as the liver, lungs and brain, and extravasation, tumor cell proliferation and angiogenesis at distal sites (5–9). Tumor cell invasion depends upon degradation of the extracellular matrix (ECM), which, when intact, acts as a barrier to block cancer cell invasion. The ECM is composed of collagen, proteoglycans, fibronectin, laminin and other glycoproteins (10–12). Two families of proteases, the matrix metalloproteinases (MMPs) and urokinase plasminogen activators (u-PA) are involved in tumor invasion and metastasis. Numerous clinical and experimental studies have demonstrated that elevated levels of u-PA and MMPs are associated with tumor growth, cancer progression, metastasis and shortened survival in patients (4,13,14).

MMPs, especially MMP-2 and MMP-9 play key roles in tumor cell invasion and metastasis due to their ability to degrade type IV collagen, a major component of the ECM (12,15,16). MMP-2 and -9 are secreted as inactive pro-enzymes in their latent zymogenic form, and activated by other MMPs or proteases. Proteolytic activities of MMP-2 and MMP-9 are inhibited by specific inhibitors, tissue inhibitors of metalloproteinases (TIMPs). Thus, a critical determinant of net proteolytic degradation is the balance between MMP and TIMP levels. Clinical studies note the association of MMP expression with progression of sarcomas (4). For example, when Benassi *et al* examined biopsied tissue immunohistochemically from patients with soft tissue sarcomas, he noted that poor prognosis was significantly correlated with elevated MMP-2 and lack of TIMP-2 expression in all sarcomas studied and that elevated MMP-2 and MMP-9 levels significantly correlated with metastasis in liposarcoma (17).

The serine protease u-PA converts plasminogen to plasmin, which is capable of promoting tumor growth and angiogenesis, degrading the ECM and basement membrane and activating pro-MMPs (18). Components of the u-PA system such as u-PA, plasminogen activator inhibitor-1 (PAI-1), and urokinase-type plasminogen activator receptor (u-PAR) are overexpressed in a variety of cancer types, most notably in breast cancer (19), but also in sarcomas (14), and correlate with cancer progression, metastasis and poor prognosis. Thus the u-PA system represents a potential target for anticancer strategies.

Rath and Pauling (20) proposed using nutrients such as lysine and ascorbic acid to target plasmin-mediated connective tissue degradation as a universal approach to tumor growth and expansion. Binding to plasminogen active sites, lysine blocks plasminogen activation into plasmin by tissue plasminogen activator (t-PA). Thus it modulates the plasmin-induced MMP activation cascade (21). Subsequent studies confirmed this approach and lead to identifying a novel formulation composed of lysine, ascorbic acid, proline and green tea extract and other micronutrients (NM), which has shown significant anticancer activity against a large number (∼40) of cancer cell lines, blocking cancer growth, tissue invasion and MMP expression both *in vitro* and *in vivo* (22). In this study, we focused on the modulating effect of NM on the activities of MMP-2 and -9, TIMPs and u-PA in adult human sarcomas: fibrosarcoma, chondrosarcoma, liposarcoma, synovial sarcoma and uterine leimyosarcoma cell lines.

## Materials and methods

### Materials

Human sarcoma cell lines fibrosarcoma HT-1080 (FS), chondrosarcoma SW-1353 (CS), liposarcoma SW-872 (LPS), synovial sarcoma SW-982 (SS) and uterine leiomyosarcoma SK-UT-1 (LS), along with their culture media were obtained from ATCC (Manassas, VA). Antibiotics, penicillin and fetal bovine serum (FBS), were obtained from Gibco-BRL (Long Island, NY). Twenty-four-well tissue culture plates were obtained from Costar (Cambridge, MA). Gelatinase zymography was performed in 10% Novex Precast SDS polyacrylamide gel (Invitrogen) with 0.1% gelatin in non-reducing conditions. The nutrient mixture (NM), prepared by VitaTech (Hayward, CA) was composed of the following ingredients in the relative amounts indicated: vitamin C (as ascorbic acid and as Mg, Ca and palmitate ascorbate) 700 mg; L-lysine 1,000 mg; L-proline 750 mg; L-arginine 500 mg; N-acetyl cysteine 200 mg; standardized green tea extract (80% polyphenol) 1,000 mg; selenium 30 *μ*g; copper 2 mg; manganese 1 mg. All other reagents used were of high quality and were obtained from Sigma, unless otherwise indicated.

### Cell cultures

The sarcoma cell lines were grown in their respective media: fibrosarcoma in MEM, chondrosarcoma in DEM, liposarcoma in MEM, synovial sarcoma in DME and uterine leiomyosarcoma in DEME, supplemented with 10% FBS, penicillin (100 U/ml) and streptomycin (100 *μ*g/ml) in 24-well tissue culture plates. The cells were plated at a density of 1×10^5^ cells/ml and grown to confluency in a humidified atmosphere at 5% CO_2_ at 37°C. Serum-supplemented media were removed and the cell monolayer was washed once with PBS with the recommended serum-free media. The cells were treated with the nutrient mixture, dissolved in media and tested at 0, 50, 100, 250, 500 and 1,000 *μ*g/ml in triplicate at each dose for u-PA and TIMP-2 studies. For MMP analysis, cells were treated with NM at 0, 10, 50, 100, 500 and 1,000 *μ*g/ml. Parallel sets of cultures were treated with PMA (100 ng/ml) for induction of MMP-9. Control and PMA treatments were done in triplicates. The plates were then returned to the incubator. The conditioned media were collected separately, pooled, and centrifuged at 4°C for 10 min at 3,000 rpm to remove cells and cell debris. The supernatant was collected and used to assess for u-PA activity (by fibrin zymography on 10% SDS-PAGE gels containing fibrinogen and plasminogen), MMP-2 and -9 (by gelatinase zymography), and TIMPs (by reverse zymography).

### Fibrin zymography

Fibrin zymography was used to analyze u-PA activity on 10% SDS-PAGE gels containing fibrinogen (5.5 mg/ml) and plasminogen (50 *μ*g/ml). After electrophoresis, the gels were washed twice with 2.5% Triton X-100 for 30 min. The gels were then incubated overnight at 37°C with 0.1% glycine buffer pH 7.5 and then stained with 0.5% Coomassie brilliant Blue R250 and destained. Electrophoresis of u-PA and t-PA were conducted for comparison. Fibrin zymograms were scanned using CanoScan 9950F Canon Scanner.

### Gelatinase zymography

Gelatinase zymography was performed in 10% Novex Precast SDS Polyacrylamide Gel (Invitrogen) in the presence of 0.1% gelatin under non-reducing conditions. Culture media (20 *μ*l) were mixed with sample buffer and loaded for SDS-PAGE with Tris-glycine SDS buffer as suggested by the manufacturer (Novex). Samples were not boiled before electrophoresis. Following electrophoresis the gels were washed twice in 2.5% Triton X-100 for 30 min at room temperature to remove SDS. The gels were then incubated at 37°C overnight in substrate buffer containing 50 mM Tris-HCl and 10 mM CaCl_2_ at pH 8.0 and stained with 0.5% Coomassie Blue R250 in 50% methanol and 10% glacial acetic acid for 30 min and destained. Upon renaturation of the enzyme, the gelatinases digest the gelatin in the gel and give clear bands against an intensely stained background. Protein standards were run concurrently and approximate molecular weights were determined by plotting the relative mobilities of known proteins.

### Reverse zymography

TIMPs were analyzed by reverse zymography on 15% SDS gels containing serum-free conditioned medium from cells. After electrophoresis the gels were washed twice with 2.5% Triton X-100 for 30 min at room temperature to remove SDS. The gels were then incubated at 37°C overnight in 50 mM Tris-HCl and 10 mM CaCl_2_ at pH 7.6 and stained with 0.5% Coomassie Blue R25, destained and scanned.

### Scanning of gelatinase, reverse and fibrin zymograms

Gelatinase, reverse and fibrin zymograms were scanned using CanoScan 9950F Canon scanner at 300 dpi. The intensity of the bands was evaluated using the pixel-based densitometer program Un-Scan-It, Version 5.1, 32-bit, by Silk Scientific Corporation (Orem, UT), at a resolution of 1 Scanner Unit (1/100 of an inch for an image that was scanned at 100 dpi). The pixel densitometer calculates the optical density of each pixel (values 0 to 255) using the darkly stained background of the gel as a pixel value of 0. A logarithmic optical density scale was used since the optical density of film and gels is logarithmically proportional to the concentration. The pixel densitometer sums the optical density of each pixel to give the band density.

### Statistical analysis

Pearson's correlation coefficient was determined between NM effect on mean MMP-2 or MMP-9, u-PA and TIMP-2 expressions of sarcoma cell lines using MedCalc Software (Mariakerke, Belgium).

## Results

Table I provides an overview of the u-PA, MMP and TIMP-2 activity in the tested sarcoma cell line.

### Effect of NM on u-PA activity in human adult sarcoma cell lines

**Figure 1.**
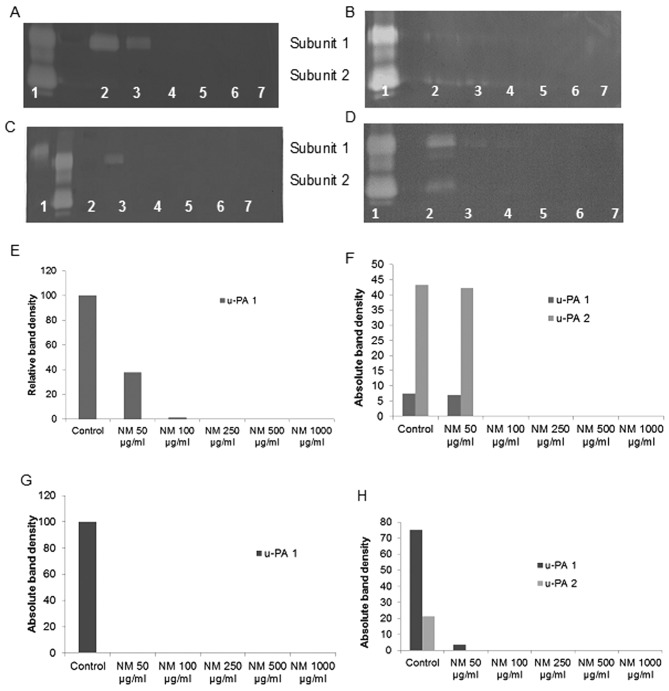
Effect of NM on fibrosarcoma HT-1080, chondrosarcoma SW-1353, liposarcoma SW-872 and leiomyosarcoma SK-UT-1 u-PA expression. Fibrin zymo-grams of (A) HT-1080, (B) SW-1353, (C) SW-872 and (D) SK-UT-1 u-PA expression. Lane 1, u-PA; lane 2, markers; lane 3, control; lanes 4–8, NM 50, 100, 250, 500 and 1,000 *μ*g/ml. Densitometric analyses of (E) HT-1080, (F) SW-1353, (G) SW-872 and (H) SK-UT-1 u-PA expression.

Activity of u-PA was detected in fibrosarcoma, chondrosarcoma, liposarcoma and uterine leiomyosarcoma cell lines. CS and LS showed 2 bands corresponding to subunits 1 and 2 at 55 and 33 kD, while FS and LPS showed one band corresponding to subunit 1 (55 kD). However, no bands corresponding to u-PA were detected for synovial sarcoma cell line. NM exerted dose response inhibition with virtual block of u-PA activity at 50 *μ*g/ml in LPS (linear trend R^2^=0.429), 100 *μ*g/ml in CS (linear trends R^2^=0.690 and 0.698 for subunits 1 and 2, respectively) and LS (linear trends R^2^=0.461 and 0.429 for subunits 1 and 2, respectively) and 250 *μ*g/ml in FS (linear trend R^2^=0.659). See Fig. 1 for respective fibrin zymograms and densitometry analyses.

### Effect of NM on MMP-2 and MMP-9 expression by fibrosarcoma cell line HT-1080

**Figure 2.**
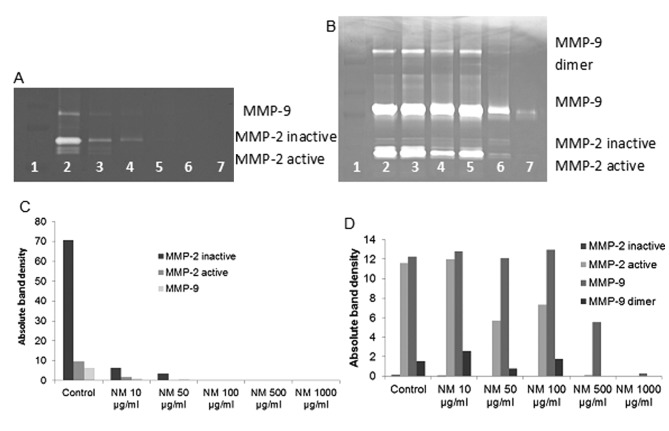
Effect of NM on fibrosarcoma HT-1080 MMP-2 and -9 expression. Gelatinase zymograms of (A) normal HT-1080 and PMA (100 ng/ml)-treated HT-1080 (B) MMP-2 and MMP-9 expression. Lane 1, markers; lane 2, control; lanes 3-7, NM 10, 50, 100, 500 and 1,000 *μ*g/ml. Densitometric analyses of (C) normal and (D) PMA-treated HT-1080 MMP-2 and -9 secretion.

On gelatinase zymography, FS cells demonstrated strong expression of MMP-2 inactive and faint MMP-2 active, both greater than MMP-9, which were inhibited by NM in a dose-dependent fashion with virtual total inhibition of MMP-9 at 100 *μ*g/ml (linear trend R^2^=0.546) and MMP-2 at 250 *μ*g/ml (linear trend R^2^=0.510). PMA (100 ng/ml) treatment enhanced MMP-2 active and MMP-9 expression by HT-108-cells; NM inhibited MMP-2 and MMP-9 in a dose-dependent manner with total block of MMP-2 active at 500 *μ*g/ml (linear trend R^2^=0.865) and near virtual block of MMP-9 at 1,000 *μ*g/ml (linear trend R^2^=0.675). See Fig. 2 for gelatinase zymograms and densitometry analyses.

### Effect of NM on MMP-2 and MMP-9 expression by chondrosarcoma cell line SW-1353

**Figure 3.**
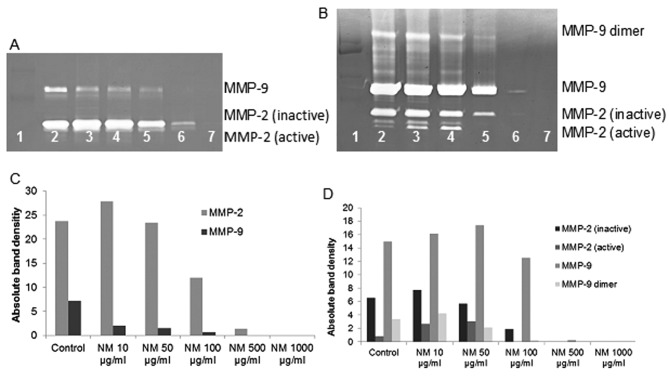
Effect of NM on chondrosarcoma SW-1353 MMP-2 and -9 expression. Gelatinase zymograms of (A) normal SW-1353 and (B) PMA-treated SW-1353 MMP-2 and MMP-9 expression. Lane 1, markers; lane 2, control; lanes 3–7, NM 10, 50, 100, 500 and 1,000 *μ*g/ml. Densitometric analyses of (C) normal and (D) PMA-treated SW-1353 MMP-2 and -9 secretion.

On gelatinase zymography, CS cells demonstrated strong expression of MMP-2 and slight expression of MMP-9, which were inhibited by NM in a dose-dependent fashion with virtual total inhibition of MMP-9 at 500 *μ*g/ml (linear trend R^2^=0.713) and MMP-2 at 1,000 *μ*g/ml (linear trend R^2^=0.860). PMA (100 ng/ml) treatment profoundly enhanced MMP-9 expression by SW-1353 cells; NM inhibited MMP-2 and MMP-9 in a dose-dependent manner with total block of MMP-2 at 500 *μ*g/ml (linear trend R^2^=0.866) and MMP-9 at 500 *μ*g/ml (linear trend R^2^=0.729). See Fig. 3 for gelatinase zymograms and densitometry analyses.

### Effect of NM on MMP-2 and MMP-9 expression by liposarcoma cell line SW-872

**Figure 4.**
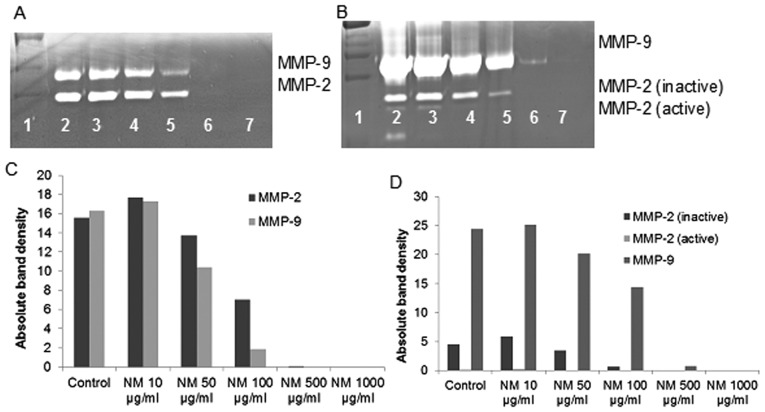
Effect of NM on liposarcoma SW-872 MMP-2 and -9 expression. Gelatinase zymograms of (A) normal SW-872 and (B) PMA-treated SW-872 MMP-2 and MMP-9 expression. Lane 1, markers; lane 2, control; lanes 3–7, NM 10, 50, 100, 500 and 1,000 *μ*g/ml. Densitometric analyses of (C) normal and (D) PMA-treated SW-872 MMP-2 and -9 secretion.

Zymography demonstrated strong expression of MMP-2 and MMP-9 by LPS cells that were inhibited by NM in a dose-dependent fashion with virtual total inhibition of MMP-2 and MMP-9 at 500 *μ*g/ml (linear trend for both R^2^=0.885). PMA (100 ng/ml) treatment profoundly enhanced MMP-9 expression by SW-872 cells; NM inhibited MMP-2 and MMP-9 in a dose-dependent manner with total block of MMP-2 at 500 *μ*g/ml (linear trend R^2^=0.821) and MMP-9 at 1,000 *μ*g/ml (linear trend R^2^=0.898). See Fig. 4 for gelatinase zymograms and densitometry analyses.

### Effect of NM on MMP-2 and MMP-9 expression by synovial sarcoma cell line SW-982

**Figure 5.**
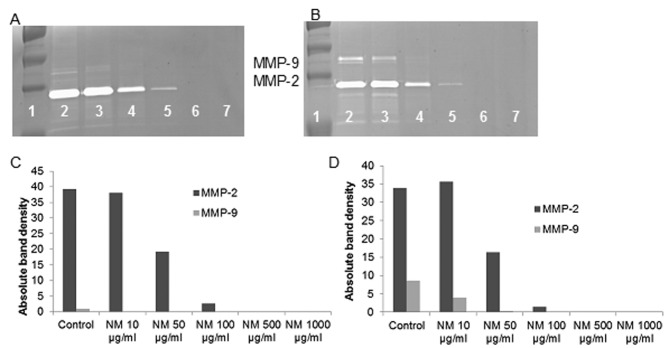
Effect of NM on synovial sarcoma SW-982 MMP-2 and -9 expression. Gelatinase zymograms of (A) normal SW-982 and (B) PMA-treated SW-872 MMP-2 and MMP-9 expression. Lane 1, markers; lane 2, control; lanes 3-7, NM 10, 50, 100, 500 and 1,000 *μ*g/ml. Densitometric analyses of (C) normal and (D) PMA-treated SW-982 MMP-2 and -9 secretion.

Zymography demonstrated secretion of MMP-2 and a faint band corresponding to MMP-9 by uninduced SS cells that were inhibited by NM in a dose-dependent fashion with virtual total inhibition of MMP-2 at 500 *μ*g/ml (linear trend R^2^=0.886) and MMP-9 at 50 *μ*g/ml (linear trend R^2^=0.429). PMA (100 ng/ml) treatment enhanced MMP-9 expression by SW-982 cells, but to a lower degree than the other cell lines; NM inhibited MMP-2 and MMP-9 in a dose-dependent manner with total block of MMP-2 at 500 *μ*g/ml (linear trend R^2^=0.855) and MMP-9 at 50 *μ*g/ml (linear trend R^2^=0.694). See Fig. 5 for gelatinase zymograms and densitometry analyses.

### Effect of NM on MMP-2 and MMP-9 expression by uterine leimyosarcoma cell line SK-UT-1 uninduced and PMA-treated

**Figure 6.**
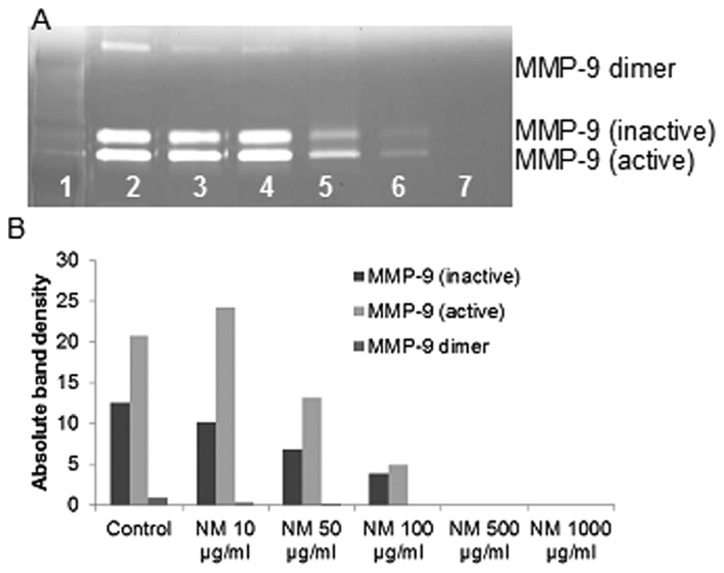
Effect of NM on uterine leiomyosarcoma SK-UT-1 MMP-9 expression. (A) Gelatinase zymogram of PMA (100 ng/ml)-treated SK-UT-1 MMP-9 expression. Lane 1, markers; lane 2, control; lanes 3–7, NM 10, 50, 100, 500 and 1,000 *μ*g/ml. (B) Densitometric analysis of PMA-treated SK-UT-1 MMP-9 secretion.

Normal LS cells did not secrete MMP-2 or MMP-9, but PMA (100 ng/ml)-treated cells demonstrated induced MMP-9 (active) greater than MMP-9 (inactive) secretions that was inhibited by NM in a dose-dependent fashion with virtual total inhibition of both at 500 *μ*g/ml (linear trends R^2^=0.884 and 0.970, respectively) See Fig. 6 for gelatinase zymogram and densitometry analysis.

### Effect of NM on TIMPs activity in fibrosarcoma, chondrosarcoma and liposarcoma

**Figure 7.**
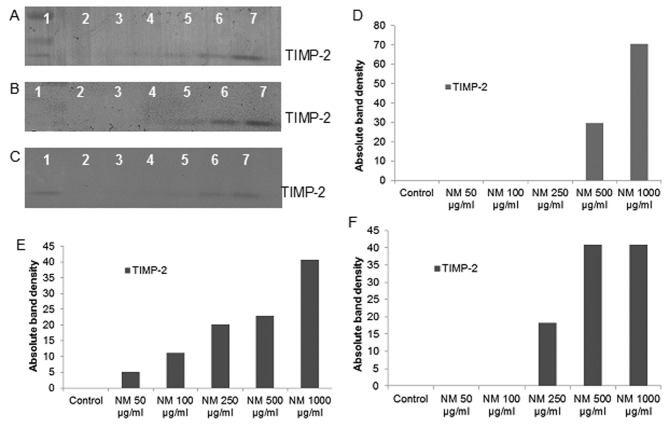
Effect of NM on fibrosarcoma HT-1080, chondrosarcoma SW-1353 and liposarcoma SW-872 TIMP-2 expression. Reverse zymograms of (A) HT-1080, (B) SW-1353 and (C) SW-872 TIMP-2 expression. Lane 1, markers; lane 2, control; lanes 3–7, NM 50, 100, 250, 500 and 1,000 *μ*g/ml. Densitometric analyses of (D) 1080, (E) SW-1353 and (F) SW-872 TIMP-2 expression.

Reverse zymography revealed upregulation of TIMP-2 activity with NM treatment in all cancer cell lines in a dose-dependent manner. Minimum activity was expressed at 50 and maximum at 1,000 *μ*g/ml NM. See Fig. 7 for respective reverse zymograms and densitometry analyses.

### Effect of NM on TIMP activity in synovial sarcoma and uterine leiomyosarcoma

**Figure 8.**
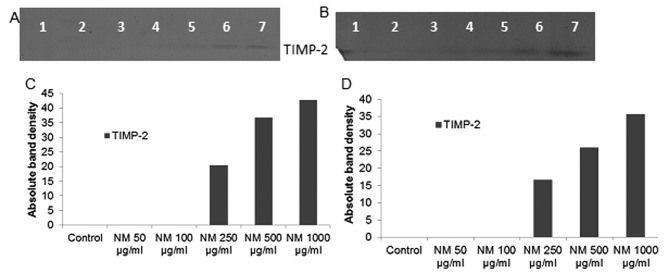
Effect of NM on synovial sarcoma SW-982 and uterine leiomyosarcoma SK-UT-1 TIMP-2 expression. Reverse zymograms of (A) SW-982 and (B) SK-UT-1 TIMP-2 expression. Lane 1, markers; lane 2, control; lanes 3-7, NM 50, 100, 250, 500 and 1,000 *μ*g/ml. Densitometric analyses of (C) SW-982 and (D) SK-UT-1 TIMP-2 expression.

Reverse zymography revealed upregulation of TIMP-2 activity with NM treatment in both sarcoma cell lines in a dose-dependent manner. Minimum activity was expressed at 50 and maximum at 1,000 *μ*g/ml NM. See Fig. 8 for respective reverse zymograms and densitometry analyses.

### Correlation between adult sarcoma u-PA, TIMP-2 and MMP expressions

**Figure 9.**
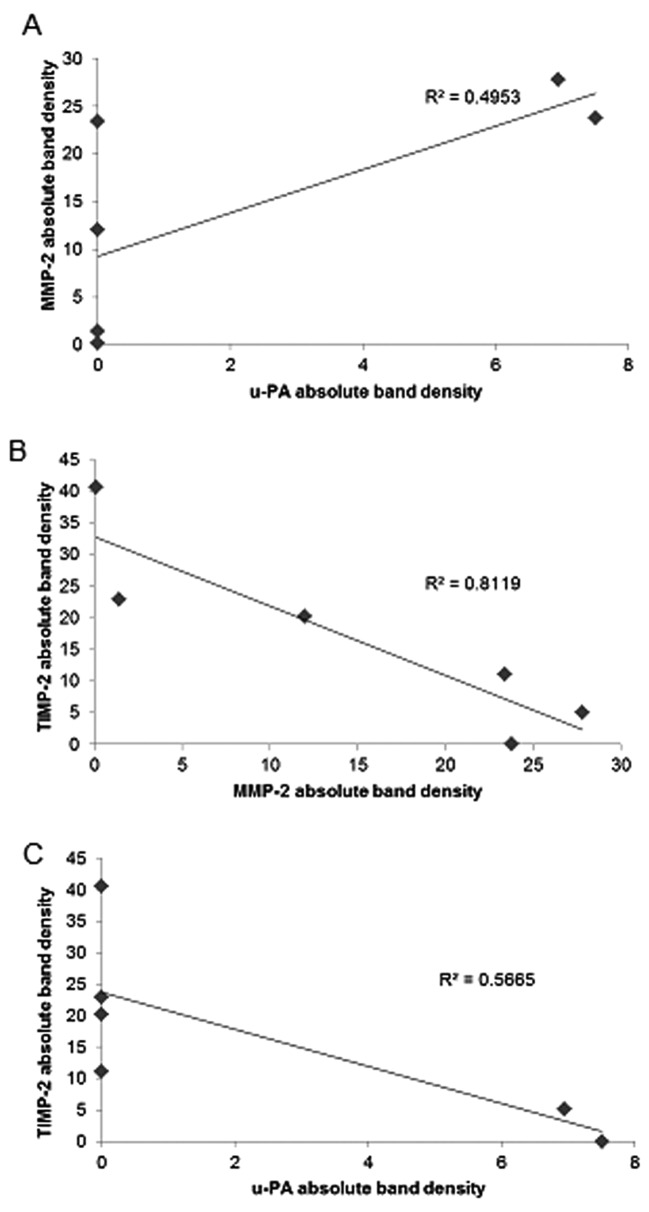
(A) Correlation between the effects of NM on chondrosarcoma SW-1353 u-PA and MMP-2 expression (correlation coefficient r=0.703). (B) Correlation between the effects of NM on chondrosarcoma SW-1353 MMP-2 and TIMP-2 expression (correlation coefficient r=−0.901). (C) Correlation between the effects of NM on chondrosarcoma SW-1353 u-PA and TIMP-2 expression (correlation coefficient r=−0.753).

Analysis revealed a positive correlation between u-PA and MMP-2 expressions of NM-treated adult sarcoma cell lines, the fibrosarcoma HT-1080, chondrosarcoma SW1353, and liposarcoma SW-872, as shown in Table II. Uterine leiomyosarcoma SK-UT-1 demonstrated a positive correlation between u-PA and MMP-9. Fig. 9A shows the correlation for chondrosarcoma u-PA and MMP-2 with a correlation coefficient r=0.703. Negative correlations were found between the expressions of MMP-2 or MMP-9 and TIMP-2 in all adult sarcoma cell lines treated with NM as shown in Table II. The correlation (r=−0.901) between MMP-2 and TIMP-2 is shown for chondrosarcoma in Fig. 9B. Negative correlations were found between expressions of TIMP-2 and u-PA in all NM-treated adult sarcoma cell lines studied, except synovial sarcoma, which did not express u-PA. The correlation (r=−0.753) between u-PA and TIMP-2 is shown for chondrosarcoma in Fig. 9C.

## Discussion

Tumor cell invasion requires the critical steps of cell attachment, degradation of the ECM and migration through the disrupted matrix. The two families of proteases, matrix metalloproteinases and urokinase plasminogen activators play key roles in tumor cell invasion. Experimental studies have demonstrated the role of urokinase plasminogen, especially cell surface u-PA, as an initiator of ECM proteolysis and associated tumor cell invasion (21). The protease u-PA converts plasminogen to plasmin, which is capable of promoting tumor growth and angiogenesis, degrading the ECM and basement membrane and activating pro-MMPs (18). Overexpression of u-PA in sarcoma patients has been correlated with cancer progression, metastasis and poor prognosis (14). Matrix metalloproteinases, especially MMP-2 and MMP-9, play pivotal roles in tumor cell invasion and metastasis due to their ability to degrade type IV collagen, a major component of the ECM. Overproduction of MMPs, especially MMP-2 and -9 and low levels of TIMPs have been shown to be associated with a more aggressive behavior of sarcomas (4,17,23).

Our study demonstrated that the specific mixture of nutrients tested significantly inhibited u-PA secretion in fibrosarcoma HT-1080, chondrosarcoma SW-1353, liposarcoma SW-872 and leiomyosarcoma SK-UT-1 cell lines (synovial sarcoma cell line SW-982 was not found to secrete u-PA in this study). Furthermore, the NM demonstrated dose-dependent decrease in MMP secretion and increase in TIMP-2 secretion by all sarcoma cell lines. As expected, a significant positive correlation was found between the secretion of u-PA and MMP-2 and a significant negative correlation between u-PA and TIMP-2 and between MMP-2 and TIMP-2 secretion by NM treatment of fibrosarcoma, chondrosarcoma and liposarcoma cells. As anticipated, a significant positive correlation was found between the secretion of u-PA and MMP-9 and a significant negative correlation was found between MMP-9 and TIMP-2 and between u-PA and TIMP-2 secretion by leiomyosarcoma SK-UT-1 cell line. Furthermore, a previous study demonstrated significant correlation between NM inhibition of Matrigel invasion and NM modulation of the MMP-2 and -9 activities of the sarcoma cell lines studied (24). A significant negative correlation was found between NM modulation of Matrigel invasion inhibition and MMP-2 secretion with fibrosarcoma HT-1080 (r=−0.911), chondrosarcoma SW-1353 (r=−0.942), liposarcoma SW-872 (r=−0.957) and synovial sarcoma SW-982 (r=−0.878). For uterine leiomyosarcoma SK-UT-1 cells, a significant negative correlation (r=−0.956) was found between NM modulation of Matrigel invasion inhibition and MMP-9 secretion. Previous *in vivo* studies of the effects of NM 0.5% dietary effect on xenograft tumor growth of fibrosarcoma and synovial sarcoma cells in nude mice support these results in that they demonstrated significant inhibition of xenograft tumor growth: 59%, p=0.0005 in fibrosarcoma HT-1080 xenografts (25) and 44%, p=0.01 in synovial sarcoma Hs 701.T xenografts (26).

In contrast to the associated toxicity and limited efficacy of standard cancer chemotherapy and radiation therapy, the efficacy and safety of dietary and botanical natural compounds in cancer prevention has been extensively documented (27). The nutrient mixture was formulated by selecting nutrients that act on critical physiological targets in cancer progression and metastasis, as documented in both clinical and experimental studies. Combining these micronutrients expands metabolic targets, maximizing biological impact with lower doses of components. A previous study of the comparative effects of NM, green tea extract and EGCG on inhibition of MMP-2 and MMP-9 secretion of different cancer cell lines with varying MMP secretion patterns, revealed the superior potency of NM over GTE and EGCG at equivalent doses (28). These results can be understood from the more comprehensive treatment offered by the combination of nutrients in NM over individual components of NM since MMP-2 and MMP-9 are mediated by differential pathways.

Optimal ECM structure depends upon adequate supplies of ascorbic acid and the amino acids lysine and proline to ensure proper synthesis and hydroxylation of collagen fibers. In addition, lysine contributes to ECM stability as a natural inhibitor of plasmin-induced proteolysis (20,29). Manganese and copper are also essential for collagen formation. There is considerable documentation of the potency of green tea extract in modulating cancer cell growth, metastasis, angiogenesis and other aspects of cancer progression (30–36). N-acetyl cysteine and selenium have demonstrated inhibition of tumor cell MMP-9 and invasive activities, as well as migration of endothelial cells through ECM (37–39). Ascorbic acid demonstrates cytotoxic and antimeta-static actions on malignant cell lines (40–44) and cancer patients have been found to have low levels of ascorbic acid (45,46). Low levels of arginine, a precursor of nitric oxide (NO), can limit the production of NO, which has been shown to predominantly act as an inducer of apoptosis (47).

In conclusion, the NM demonstrated potent anticancer activity by targeting primary mechanisms responsible for the aggressive spread of adult sarcomas. In this *in vitro* study, the NM significantly inhibited secretion of u-PA and MMP-2 and/or -9 and increased secretion of TIMP-2 in fibrosarcoma, chondrosarcoma, liposarcoma and uterine leiomyosarcoma cells, suggesting its potential in modulating cancer invasion and metastasis. Malignant synovial sarcoma cells did not secrete u-PA; however, MMP-2 and -9 secretions by SW-982 were inhibited by NM and secretion of TIMP-2 was enhanced by NM. NM inhibition of MMP secretion was found to be correlated significantly with Matrigel invasion of all the sarcoma cell lines studied. Furthermore, use of the nutrient mixture would not pose any toxic effect clinically, especially in the relevant doses, as *in vivo* safety studies demonstrate. An *in vivo* toxicology study showed that NM had no adverse effects on vital organs (heart, liver and kidney), or on the associated functional serum enzymes (48).

## Figures and Tables

**Table I.** Overview of MMP-2 and -9, u-PA and TIMP-2 expression of adult sarcoma cell lines.

Cancer cell line	MMP-2	MMP-9	u-PA	TIMP-2
Fibrosarcoma HT-1080	+	+	+	+
Chondrosarcoma SW-1353	+	+	+	+
Liposarcoma SW-872	+	+	+	+
Synovial sarcoma SW-982	+	+	−	+
Uterine leiomyosarcoma SK-UT-1	−	+	+	+

**Table II.** Correlation between effects of NM on sarcoma cell u-PA, MMPs and TIMPs.

Cell line	u-PA and MMPs	MMPs and TIMPs	u-PA and TIMPs
Fibrosarcoma HT-1080	r=0.974	r=−0.360	r=−0.397
Chondrosarcoma SW-1353	r=0.703	r=−0.901	r=−0.753
Liposarcoma SW-872	r=0.412	r=−0.983	r=−0.407
Synovial sarcoma SW-982	N/A	r=−0.861	N/A
Uterine leiomyosarcoma SK-UT-1	r=0.683	r=−0.917	r=−0.436
